# Poly[bis­(μ_4_-oxalato)potassium(I)praseodymium(III)]

**DOI:** 10.1107/S2414314625006078

**Published:** 2025-07-11

**Authors:** Kanthida Kummoon, Sakchai Laksee, Kittipong Chainok

**Affiliations:** a Thammasat University Research Unit in Multifunctional Crystalline Materials and Applications (TU-McMa) Faculty of Science and Technology Thammasat University Pathum Thani 12121 Thailand; b Nuclear Technology Research and Development Center, Thailand Institute of Nuclear Technology (Public Organization), Nakhon Nayok 26120, Thailand; Vienna University of Technology, Austria

**Keywords:** crystal structure, coordination polymer, oxalate, potassium, praseodymium

## Abstract

The asymmetric unit of the title oxalate-bridged bimetallic coordination polymer, [KPr(C_2_O_4_)_2_]_*n*_, contains one Pr^3+^ cation, one K^+^ cation, one complete C_2_O_4_^2–^ anion, and one half of each of two C_2_O_4_^2–^ anions positioned on crystallographic inversion centers in the monoclinic space group *P*2_1_/*c*. The completely deprotonated C_2_O_4_^2–^ ligands exhibit a *μ*_4_-chelating/bridging coordination mode that connects the Pr^3+^ and K^+^ cations into a framework structure.

## Abstract 2

The oxalate-bridged alkali/lanthanide bimetallic coordination polymer is formed by linking Pr^3+^ and K^+^ cations atoms through oxalate ligands using a *μ*_4_-chelating/bridging coordination mode.

## Graphical abstract

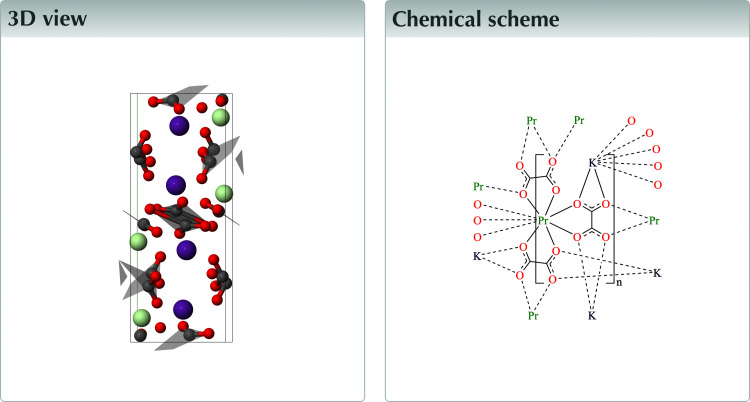

## Structure description

For over a decade, lanthanide-based coordination polymers have garnered significant inter­est due to their intriguing structural topologies and prospective applications in gas storage, catalysis, separation, luminescence or mol­ecular magnetism (Patra & Pal, 2025 ▸; Wang *et al.*, 2025 ▸; Zhang *et al.*, 2021 ▸). Because of the strong Lewis acidity of lanthanide ions as hard Pearson acids, ligands featuring donor oxygen atoms have been thoroughly investigated. Likewise, polycarboxyl­ate ligands have been attracting inter­est due to their chemical and thermal stability, capacity to affect structural details *via* hydrogen-bonding inter­actions, and carboxyl­ate functional groups that provide extensive structural diversity through several possible coordination modes (Janicki *et al.*, 2017 ▸; Liu *et al.*, 2010 ▸). For the current study, oxalate (C_2_O_4_^2–^) ligands were employed to synthesize novel bimetallic coordination polymers based on specific rational designs. The oxalate anion has four oxygen atoms capable of coordinating to lanthanide cations in several coordination modes. In addition, alkali metal cations were incorporated with the premise that the synergistic inter­actions between alkali and lanthanide metal ions might promote the formation of novel heterometallic coordination polymers exhibiting new crystal structures (Ponjan *et al.*, 2020 ▸). A search in the Cambridge Structural Database (CSD, version 5.46, last update February 2025; Groom *et al.*, 2016 ▸) using the CONQUEST software (Bruno *et al.*, 2002 ▸) revealed that only two crystal structures of oxalate-bridged coordination polymers containing potassium(I) and praseodymium(III) ions are documented: [K_2_Pr_2_(C_2_O_4_)_4_(H_2_O)] (COYNOV; Hong *et al.*, 2014 ▸) and [KPr_2_(C_2_O_4_)_0.5_(C_8_H_4_O_4_)_3_(H_2_O)_3_] (NULYOJ; Yang *et al.*, 2009 ▸). In the current data report, we present the synthesis and crystal structure of a novel oxalate-bridged potassium(I)-praseodymium(III) heterometallic complex, [KPr(C_2_O_4_)_2_]_*n*_.

**Figure 1:**
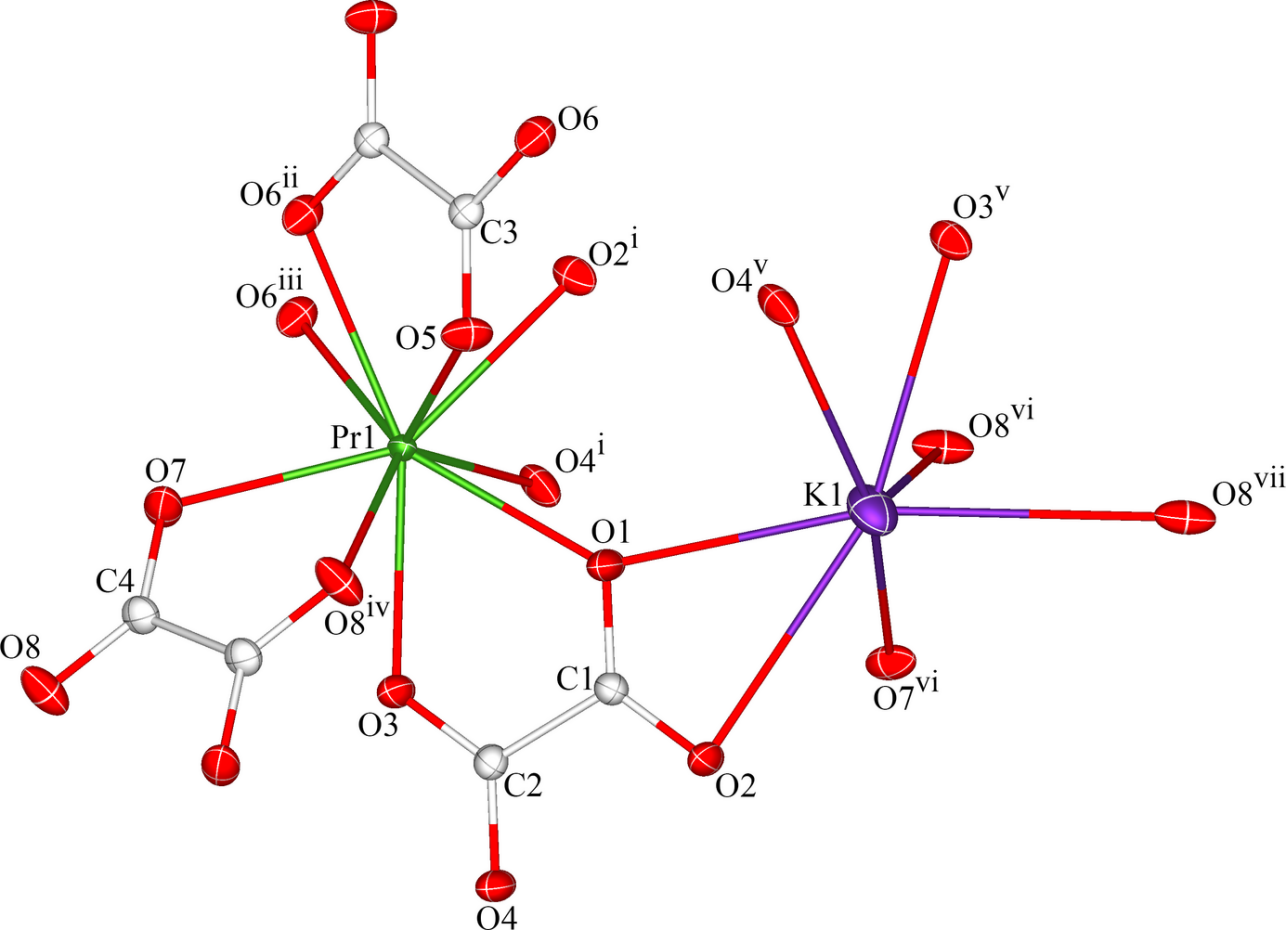
The enlarged asymmetric unit of the title coordination polymer illustrating the complete coordination spheres of the Pr^3+^ and K^+^ cations. Displacement ellipsoids are depicted at the 50% probability level. [Symmetry codes: (i) *x*, 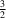 − *y*, *z* − 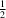; (ii) 1 − *x*, 1 − *y*, −*z*; (iii) 1 + *x*, *y*, *z*; (iv) 2 − *x*, 1 − *y*, 1 − *z*; (v) *x* − 1, 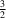 − *y*, *z* − 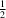; (vi) 2 − *x*, 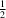 + *y*, 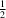 − *z*; (vii) *x* − 1, 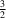 − *y*, *z* − 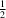.]

**Figure 2:**
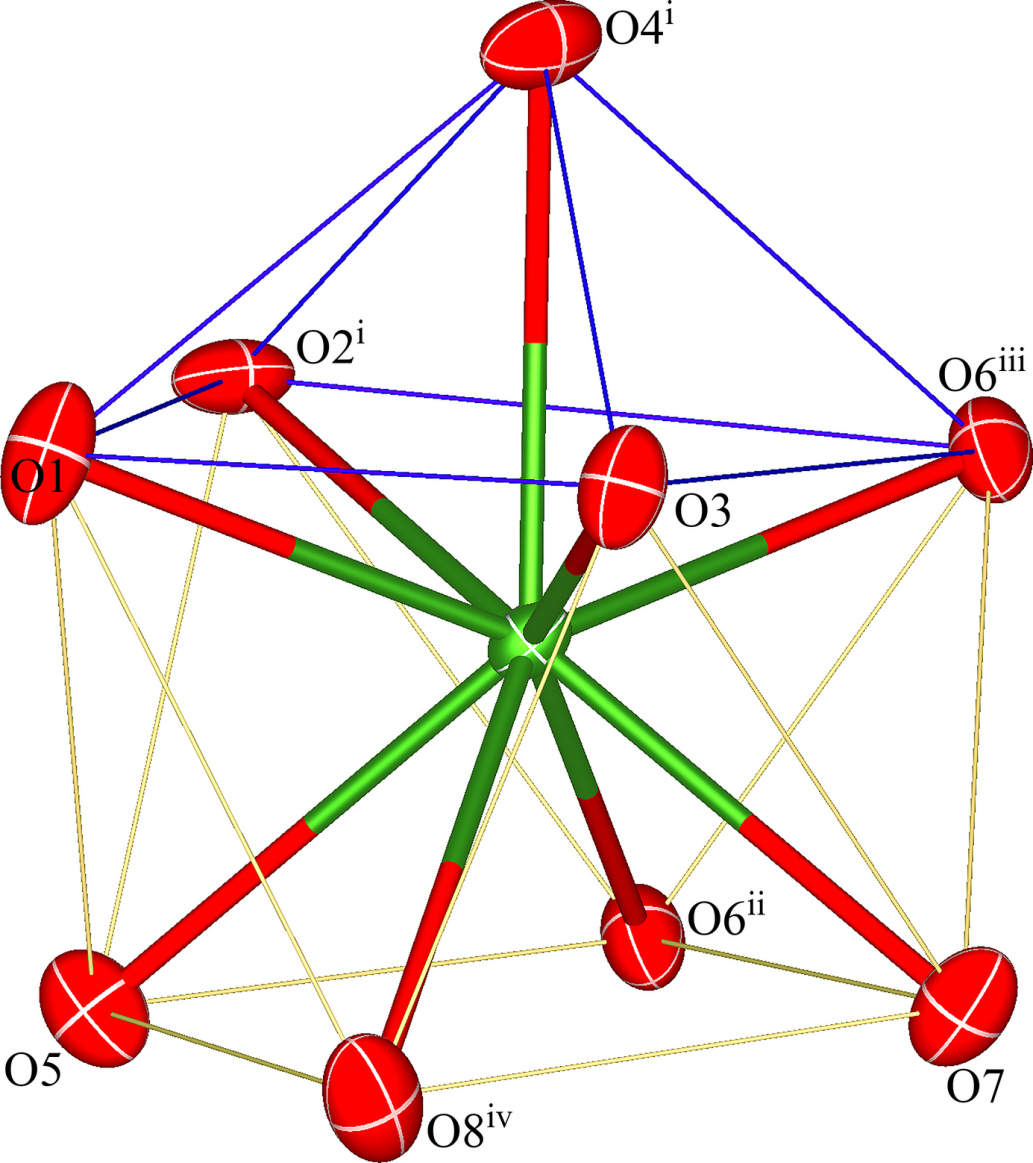
Coordination polyhedron around the Pr^3+^ cation in the title coordination polymer. Symmetry codes and displacement ellipsoids are as in Fig. 1 ▸.

The asymmetric unit of the title coordination polymer comprises one Pr^3+^ cation, one K^+^ cation, one complete C_2_O_4_^2–^ anion, and two half of each of two C_2_O_4_^2–^ anions situated at crystallographic inversion centers. Fig. 1 ▸ shows the ninefold coordination of the Pr^3+^ cation by nine oxygen atoms from five distinct C_2_O_4_^2–^ ligands with the O4^i^ atom [symmetry code (i): *x*, 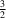 − *y*, *z* − 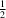] occupying the capping position of the distorted monocapped anti­prism (Fig. 2 ▸). The Pr—O bond lengths vary from 2.4590 (17) to 2.6011 (18) Å, with an average bond length of 2.528 Å. The K^+^ cation is coordinated by seven oxygen atoms from four different C_2_O_4_^2–^ ligands. The K—O bond lengths range from 2.7664 (18) to 3.152 (2) Å, with an average bond length of 2.919 Å. The bond lengths in the two metal–oxygen polyhedra are comparable to those for the related compounds mentioned above (Hong *et al.*, 2014 ▸; Yang *et al.*, 2009 ▸).

**Figure 3:**
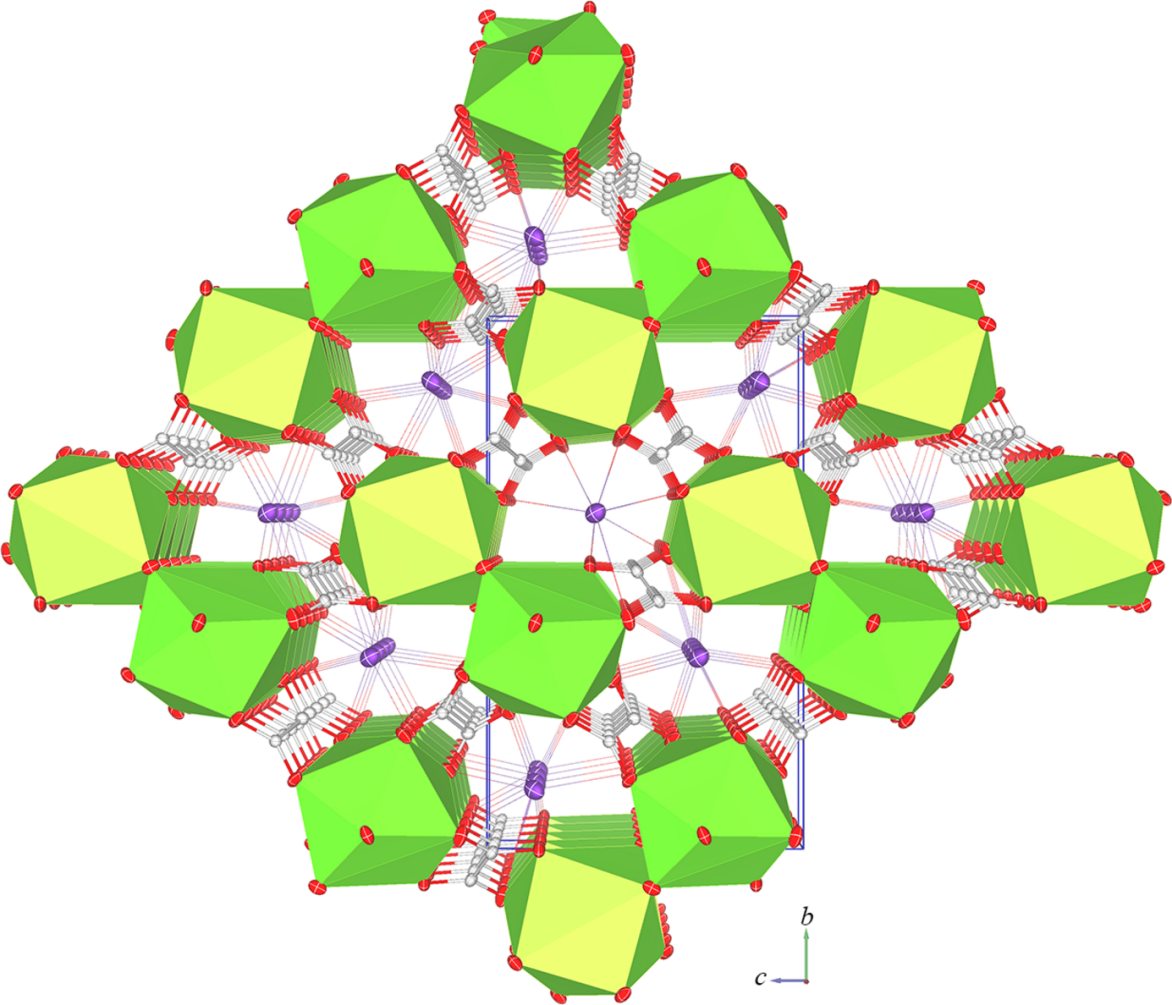
Perspective view of the framework structure along the *a* axis containing the coordination polyhedron of the Pr^3+^ cation. The K—O bonds are represented by small rods. Displacement ellipsoids are as in Fig. 1 ▸.

The oxalate anion, C1/C2/O1/O2/O3/O4, which is situated in a general position, coordinates to the central Pr^3+^ and K^+^ cations in a *μ*_4_-κ^2^*O*1,*O*2:κ^2^*O*3,*O*4:κ^2^*O*1,*O*3:κ^2^*O*2,*O*4 coordination mode, leading to the formation of a corrugated sheet extending in the *ac* plane. Neighbouring sheets are linked by the two other C_2_O_4_^2–^ ligands positioned over inversion centers, C3/C3^ii^/O5/O5^ii^/O6/O6^ii^ [symmetry code: (ii) −*x* + 1, −*y* + 1, −*z*] and C4/C4^iv^/O7/O7^iv^/O8/O8^iv^ [symmetry code (iv): −*x* + 2, −*y* + 1, −*z* + 1], in a *μ*_4_-κ*O*6:κ*O*6^ii^:κ^2^*O*5,*O*6^ii^:κ^2^*O*5^ii^,*O*6 and *μ*_4_-κ^2^*O*7,*O*8:κ^2^*O*7^iv^′,*O*8^iv^:κ^2^*O*7,*O*8^iv^:κ^2^*O*7^iv^*O*8 coordination mode, respectively, creating a tri-periodic framework structure (Fig. 3 ▸).

## Synthesis and crystallization

A mixture of Pr(NO_3_)_3_·6H_2_O (0.218 g, 0.5 mmol), oxalic acid (0.045 g, 0.5 mmol) and KOH (0.112 g, 2.0 mmol) in a mixed water (5 ml) and DMF (5 ml) solution was sealed in a 23 ml Teflon-lined steel autoclave and heated at 463 K for 48 h. The autoclave was then cooled to room temperature, and light-green hexa­gonal-shaped crystals were obtained in a yield of 57% (0.124 g) based on Pr(NO_3_)_3_·6H_2_O.

## Refinement

Crystal data, data collection and structure refinement details are summarized in Table 1 ▸.

## Supplementary Material

Crystal structure: contains datablock(s) I. DOI: 10.1107/S2414314625006078/wm4230sup1.cif

Structure factors: contains datablock(s) I. DOI: 10.1107/S2414314625006078/wm4230Isup2.hkl

Supporting information file. DOI: 10.1107/S2414314625006078/wm4230Isup3.cdx

Letter of Response. DOI: 10.1107/S2414314625006078/wm4230sup4.pdf

CCDC reference: 2470468

Additional supporting information:  crystallographic information; 3D view; checkCIF report

## Figures and Tables

**Table 1:** Experimental details

Crystal data	
Chemical formula	[KPr(C_2_O_4_)_2_]
*M*_r_	356.05
Crystal system, space group	Monoclinic, *P*2_1_/*c*
Temperature (K)	296
*a*, *b*, *c* (Å)	5.7205 (1), 14.9416 (3), 8.8848 (2)
β (°)	92.665 (1)
*V* (Å^3^)	758.59 (3)
*Z*	4
Radiation type	Mo *K*α
μ (mm^−1^)	6.99
Crystal size (mm)	0.12 × 0.06 × 0.06

Data collection	
Diffractometer	Bruker D8 QUEST CMOS PHOTON II
Absorption correction	Multi-scan (*SADABS*; Krause *et al.*, 2015 ▸)
*T*_min_, *T*_max_	0.603, 0.746
No. of measured, independent and observed [*I* > 2σ(*I*)] reflections	18857, 1889, 1829
*R*_int_	0.032
(sin θ/λ)_max_ (Å^−1^)	0.668

Refinement	
*R*[*F*^2^ > 2σ(*F*^2^)], *wR*(*F*^2^), *S*	0.016, 0.038, 1.20
No. of reflections	1889
No. of parameters	127
Δρ_max_, Δρ_min_ (e Å^−3^)	0.44, −1.07

Computer programs: *APEX6* and *SAINT* (Bruker, 2023 ▸), *SHELXT* (Sheldrick, 2015*a* ▸), *SHELXL* (Sheldrick, 2015*b* ▸) and *OLEX2* (Dolomanov *et al.*, 2009 ▸).

## full crystallographic data

### Poly[bis(µ4-oxalato)potassium(I)praseodymium(III)] Crystal data

**Table:**

[KPr(C_2_O_4_)_2_]	*F*(000) = 664
*M**_r_* = 356.05	*D*_x_ = 3.118 Mg m^−^^3^
Monoclinic, *P*2_1_/*c*	Mo *K*α radiation, λ = 0.71073 Å
*a* = 5.7205 (1) Å	Cell parameters from 9930 reflections
*b* = 14.9416 (3) Å	θ = 2.7–28.3°
*c* = 8.8848 (2) Å	µ = 6.99 mm^−^^1^
β = 92.665 (1)°	*T* = 296 K
*V* = 758.59 (3) Å^3^	Hexagonal, light green
*Z* = 4	0.12 × 0.06 × 0.06 mm

### Poly[bis(µ4-oxalato)potassium(I)praseodymium(III)] Data collection

**Table:**

BRUKER D8 QUEST CMOS PHOTON II diffractometer	1889 independent reflections
Radiation source: sealed x-ray tube	1829 reflections with *I* > 2σ(*I*)
Graphite monochromator	*R*_int_ = 0.032
Detector resolution: 7.39 pixels mm^-1^	θ_max_ = 28.4°, θ_min_ = 2.7°
ω and φ scans	*h* = −7→7
Absorption correction: multi-scan (SADABS; Krause *et al.*, 2015)	*k* = −19→19
*T*_min_ = 0.603, *T*_max_ = 0.746	*l* = −11→11
18857 measured reflections	

### Poly[bis(µ4-oxalato)potassium(I)praseodymium(III)] Refinement

**Table:**

Refinement on *F*^2^	0 restraints
Least-squares matrix: full	Primary atom site location: dual
*R*[*F*^2^ > 2σ(*F*^2^)] = 0.016	*w* = 1/[σ^2^(*F*_o_^2^) + (0.0178*P*)^2^ + 0.5554*P*] where *P* = (*F*_o_^2^ + 2*F*_c_^2^)/3
*wR*(*F*^2^) = 0.038	(Δ/σ)_max_ = 0.001
*S* = 1.20	Δρ_max_ = 0.44 e Å^−^^3^
1889 reflections	Δρ_min_ = −1.07 e Å^−^^3^
127 parameters	

### Poly[bis(µ4-oxalato)potassium(I)praseodymium(III)] Special details

**Table:**

Geometry. All esds (except the esd in the dihedral angle between two l.s. planes) are estimated using the full covariance matrix. The cell esds are taken into account individually in the estimation of esds in distances, angles and torsion angles; correlations between esds in cell parameters are only used when they are defined by crystal symmetry. An approximate (isotropic) treatment of cell esds is used for estimating esds involving l.s. planes.

### Poly[bis(µ4-oxalato)potassium(I)praseodymium(III)] Fractional atomic coordinates and isotropic or equivalent isotropic displacement parameters (Å^2^)

**Table:**

	*x*	*y*	*z*	*U*_iso_*/*U*_eq_	
K1	0.45578 (11)	0.86950 (4)	0.15682 (7)	0.02952 (13)	
Pr1	0.92864 (2)	0.59670 (2)	0.18326 (2)	0.01100 (5)	
O1	0.7504 (3)	0.73622 (12)	0.2696 (2)	0.0226 (4)	
O2	0.7493 (3)	0.84258 (11)	0.44375 (19)	0.0190 (3)	
O3	1.1506 (3)	0.66905 (11)	0.39824 (19)	0.0190 (3)	
O4	1.1438 (3)	0.77457 (12)	0.5752 (2)	0.0214 (4)	
O5	0.4910 (3)	0.56301 (12)	0.16446 (19)	0.0205 (4)	
O6	0.2083 (3)	0.53450 (11)	−0.0105 (2)	0.0182 (3)	
O7	1.1044 (3)	0.46803 (12)	0.32627 (18)	0.0216 (4)	
O8	1.2497 (3)	0.44223 (14)	0.56080 (19)	0.0245 (4)	
C1	0.8346 (4)	0.77504 (15)	0.3838 (3)	0.0139 (4)	
C2	1.0638 (4)	0.73668 (15)	0.4589 (3)	0.0143 (4)	
C3	0.4158 (4)	0.52864 (14)	0.0453 (2)	0.0128 (4)	
C4	1.1034 (4)	0.47399 (15)	0.4679 (3)	0.0159 (4)	

### Poly[bis(µ4-oxalato)potassium(I)praseodymium(III)] Atomic displacement parameters (Å^2^)

**Table:**

	*U*^11^	*U*^22^	*U*^33^	*U*^12^	*U*^13^	*U*^23^
K1	0.0228 (3)	0.0313 (3)	0.0336 (3)	−0.0036 (2)	−0.0085 (2)	0.0047 (3)
Pr1	0.01561 (8)	0.00942 (7)	0.00784 (7)	0.00076 (4)	−0.00097 (5)	−0.00028 (4)
O1	0.0259 (10)	0.0200 (8)	0.0208 (9)	0.0084 (7)	−0.0103 (7)	−0.0087 (7)
O2	0.0222 (9)	0.0178 (8)	0.0166 (8)	0.0076 (7)	−0.0035 (7)	−0.0061 (6)
O3	0.0209 (9)	0.0185 (8)	0.0172 (8)	0.0055 (7)	−0.0043 (7)	−0.0058 (7)
O4	0.0229 (9)	0.0196 (8)	0.0208 (9)	0.0052 (7)	−0.0085 (7)	−0.0082 (7)
O5	0.0193 (9)	0.0271 (9)	0.0152 (8)	−0.0018 (7)	0.0018 (7)	−0.0071 (7)
O6	0.0127 (8)	0.0172 (8)	0.0247 (9)	0.0004 (6)	0.0002 (7)	−0.0049 (7)
O7	0.0329 (10)	0.0201 (9)	0.0119 (8)	0.0076 (7)	0.0017 (7)	0.0005 (6)
O8	0.0195 (9)	0.0383 (11)	0.0158 (8)	0.0070 (8)	−0.0001 (7)	0.0072 (8)
C1	0.0157 (11)	0.0129 (10)	0.0130 (10)	−0.0001 (8)	−0.0016 (8)	0.0001 (8)
C2	0.0152 (11)	0.0123 (10)	0.0153 (11)	−0.0006 (8)	−0.0001 (8)	0.0002 (8)
C3	0.0133 (10)	0.0121 (10)	0.0132 (10)	−0.0017 (8)	0.0026 (8)	0.0011 (8)
C4	0.0199 (12)	0.0152 (10)	0.0129 (10)	−0.0015 (9)	0.0011 (8)	0.0032 (8)

### Poly[bis(µ4-oxalato)potassium(I)praseodymium(III)] Geometric parameters (Å, º)

**Table:**

K1—O1	2.7664 (18)	Pr1—O7	2.4905 (18)
K1—O2	3.0138 (18)	Pr1—O8^vi^	2.6011 (18)
K1—O3^i^	2.8785 (18)	O1—C1	1.246 (3)
K1—O4^i^	2.8679 (18)	O2—C1	1.251 (3)
K1—O7^ii^	2.9131 (19)	O3—C2	1.258 (3)
K1—O8^ii^	2.8392 (19)	O4—C2	1.247 (3)
K1—O8^i^	3.152 (2)	O5—C3	1.236 (3)
Pr1—O1	2.4590 (17)	O6—C3	1.268 (3)
Pr1—O2^iii^	2.4901 (17)	O7—C4	1.262 (3)
Pr1—O3	2.4906 (17)	O8—C4	1.242 (3)
Pr1—O4^iii^	2.4990 (17)	C1—C2	1.553 (3)
Pr1—O5	2.5514 (18)	C3—C3^iv^	1.543 (4)
Pr1—O6^iv^	2.5883 (16)	C4—C4^vi^	1.547 (5)
Pr1—O6^v^	2.5777 (17)		
			
O1—K1—O2	44.88 (5)	O7—Pr1—O5	104.63 (6)
O1—K1—O3^i^	118.80 (5)	O7—Pr1—O6^v^	79.29 (5)
O1—K1—O4^i^	84.99 (6)	O7—Pr1—O6^iv^	79.73 (6)
O1—K1—O7^ii^	80.50 (6)	O7—Pr1—O8^vi^	63.05 (6)
O1—K1—O8^ii^	98.71 (6)	Pr1—O1—K1	138.75 (7)
O1—K1—O8^i^	162.61 (6)	C1—O1—Pr1	119.90 (15)
O2—K1—O8^i^	122.32 (5)	C1—O1—K1	99.54 (13)
O3^i^—K1—O2	160.37 (5)	Pr1^vii^—O2—K1	149.93 (7)
O3^i^—K1—O7^ii^	129.25 (5)	C1—O2—Pr1^vii^	120.31 (14)
O3^i^—K1—O8^i^	75.95 (5)	C1—O2—K1	87.73 (13)
O4^i^—K1—O2	115.43 (5)	Pr1—O3—K1^viii^	142.55 (7)
O4^i^—K1—O3^i^	45.63 (5)	C2—O3—Pr1	118.69 (14)
O4^i^—K1—O7^ii^	156.68 (6)	C2—O3—K1^viii^	93.18 (13)
O4^i^—K1—O8^i^	112.39 (5)	Pr1^vii^—O4—K1^viii^	142.41 (7)
O7^ii^—K1—O2	65.03 (5)	C2—O4—Pr1^vii^	119.98 (15)
O7^ii^—K1—O8^i^	82.78 (5)	C2—O4—K1^viii^	93.94 (14)
O8^ii^—K1—O2	107.59 (5)	Pr1—O5—K1^ix^	94.19 (5)
O8^ii^—K1—O3^i^	83.70 (5)	C3—O5—Pr1	116.08 (15)
O8^ii^—K1—O4^i^	119.81 (6)	C3—O5—K1^ix^	93.93 (14)
O8^ii^—K1—O7^ii^	45.83 (5)	Pr1^x^—O6—Pr1^iv^	119.27 (6)
O8^ii^—K1—O8^i^	72.57 (6)	C3—O6—Pr1^iv^	115.59 (14)
O1—Pr1—O2^iii^	78.31 (6)	C3—O6—Pr1^x^	111.22 (14)
O1—Pr1—O3	66.22 (6)	Pr1—O7—K1^xi^	138.04 (7)
O1—Pr1—O4^iii^	71.70 (7)	C4—O7—Pr1	115.73 (15)
O1—Pr1—O5	76.58 (6)	C4—O7—K1^xi^	91.60 (14)
O1—Pr1—O6^iv^	135.11 (6)	Pr1^vi^—O8—K1^viii^	96.89 (6)
O1—Pr1—O6^v^	142.50 (6)	Pr1^vi^—O8—K1^xi^	141.18 (8)
O1—Pr1—O7	131.20 (6)	K1^xi^—O8—K1^viii^	107.43 (6)
O1—Pr1—O8^vi^	74.44 (6)	C4—O8—Pr1^vi^	112.76 (15)
O2^iii^—Pr1—O3	132.24 (6)	C4—O8—K1^xi^	95.54 (14)
O2^iii^—Pr1—O4^iii^	65.29 (5)	C4—O8—K1^viii^	94.06 (15)
O2^iii^—Pr1—O5	69.64 (6)	O1—C1—O2	125.4 (2)
O2^iii^—Pr1—O6^v^	78.71 (6)	O1—C1—C2	117.7 (2)
O2^iii^—Pr1—O6^iv^	70.61 (5)	O2—C1—C2	116.9 (2)
O2^iii^—Pr1—O7	149.06 (6)	O3—C2—K1^viii^	63.74 (12)
O2^iii^—Pr1—O8^vi^	131.75 (6)	O3—C2—C1	117.0 (2)
O3—Pr1—O4^iii^	73.62 (6)	O4—C2—K1^viii^	63.23 (12)
O3—Pr1—O5	126.83 (6)	O4—C2—O3	125.7 (2)
O3—Pr1—O6^v^	111.02 (6)	O4—C2—C1	117.3 (2)
O3—Pr1—O6^iv^	155.90 (6)	C1—C2—K1^viii^	166.83 (15)
O3—Pr1—O8^vi^	68.48 (6)	O5—C3—O6	126.1 (2)
O4^iii^—Pr1—O5	128.75 (6)	O5—C3—C3^iv^	118.5 (2)
O4^iii^—Pr1—O6^iv^	119.74 (6)	O6—C3—C3^iv^	115.4 (2)
O4^iii^—Pr1—O6^v^	71.88 (6)	K1^xi^—C4—K1^viii^	92.69 (6)
O4^iii^—Pr1—O8^vi^	136.72 (6)	O7—C4—K1^xi^	65.24 (13)
O5—Pr1—O6^iv^	62.79 (5)	O7—C4—K1^viii^	120.05 (16)
O5—Pr1—O6^v^	121.51 (5)	O7—C4—C4^vi^	116.2 (3)
O5—Pr1—O8^vi^	65.77 (6)	O8—C4—K1^viii^	65.02 (14)
O6^v^—Pr1—O6^iv^	60.73 (6)	O8—C4—K1^xi^	61.79 (13)
O6^iv^—Pr1—O8^vi^	103.22 (6)	O8—C4—O7	127.0 (2)
O6^v^—Pr1—O8^vi^	141.64 (6)	O8—C4—C4^vi^	116.8 (2)
O7—Pr1—O3	76.38 (6)	C4^vi^—C4—K1^viii^	85.41 (17)
O7—Pr1—O4^iii^	126.57 (6)	C4^vi^—C4—K1^xi^	178.0 (2)
			
K1—O1—C1—O2	17.8 (3)	Pr1—O3—C2—C1	−5.8 (3)
K1—O1—C1—C2	−162.38 (17)	Pr1^vii^—O4—C2—K1^viii^	−163.10 (16)
K1—O2—C1—O1	−16.1 (3)	Pr1^vii^—O4—C2—O3	−176.62 (18)
K1—O2—C1—C2	164.10 (18)	Pr1^vii^—O4—C2—C1	2.1 (3)
K1^viii^—O3—C2—O4	13.5 (3)	Pr1—O5—C3—O6	−153.70 (19)
K1^viii^—O3—C2—C1	−165.28 (17)	Pr1—O5—C3—C3^iv^	27.5 (3)
K1^viii^—O4—C2—O3	−13.5 (3)	Pr1^x^—O6—C3—O5	−13.3 (3)
K1^viii^—O4—C2—C1	165.21 (17)	Pr1^iv^—O6—C3—O5	−153.45 (19)
K1^ix^—O5—C3—O6	109.7 (2)	Pr1^iv^—O6—C3—C3^iv^	25.4 (3)
K1^ix^—O5—C3—C3^iv^	−69.1 (2)	Pr1^x^—O6—C3—C3^iv^	165.52 (19)
K1^xi^—O7—C4—K1^viii^	78.04 (13)	Pr1—O7—C4—K1^xi^	−147.06 (14)
K1^xi^—O7—C4—O8	−1.9 (3)	Pr1—O7—C4—K1^viii^	−69.02 (18)
K1^xi^—O7—C4—C4^vi^	178.5 (2)	Pr1—O7—C4—O8	−148.9 (2)
K1^viii^—O8—C4—K1^xi^	107.97 (7)	Pr1—O7—C4—C4^vi^	31.5 (3)
K1^xi^—O8—C4—K1^viii^	−107.97 (7)	Pr1^vi^—O8—C4—K1^xi^	−152.81 (15)
K1^xi^—O8—C4—O7	1.9 (3)	Pr1^vi^—O8—C4—K1^viii^	99.21 (11)
K1^viii^—O8—C4—O7	109.9 (2)	Pr1^vi^—O8—C4—O7	−150.9 (2)
K1^xi^—O8—C4—C4^vi^	−178.5 (2)	Pr1^vi^—O8—C4—C4^vi^	28.7 (3)
K1^viii^—O8—C4—C4^vi^	−70.5 (3)	O1—C1—C2—K1^viii^	−89.4 (7)
Pr1—O1—C1—O2	−174.77 (18)	O1—C1—C2—O3	0.6 (3)
Pr1—O1—C1—C2	5.0 (3)	O1—C1—C2—O4	−178.3 (2)
Pr1^vii^—O2—C1—O1	175.37 (19)	O2—C1—C2—K1^viii^	90.4 (7)
Pr1^vii^—O2—C1—C2	−4.5 (3)	O2—C1—C2—O3	−179.6 (2)
Pr1—O3—C2—K1^viii^	159.52 (15)	O2—C1—C2—O4	1.6 (3)
Pr1—O3—C2—O4	172.99 (19)		

Symmetry codes: (i) *x*−1, −*y*+3/2, *z*−1/2; (ii) −*x*+2, *y*+1/2, −*z*+1/2; (iii) *x*, −*y*+3/2, *z*−1/2; (iv) −*x*+1, −*y*+1, −*z*; (v) *x*+1, *y*, *z*; (vi) −*x*+2, −*y*+1, −*z*+1; (vii) *x*, −*y*+3/2, *z*+1/2; (viii) *x*+1, −*y*+3/2, *z*+1/2; (ix) −*x*+1, *y*−1/2, −*z*+1/2; (x) *x*−1, *y*, *z*; (xi) −*x*+2, *y*−1/2, −*z*+1/2.
