# Supplementary material for: Poly[bis­(μ4-oxalato)potassium(I)praseodymium(III)]
Source: IUCrdata. 2025 Jul 11;10(Pt 7):x250607. doi: 10.1107/S2414314625006078 (PMC12326465; doi:10.1107/S2414314625006078)
Supplement: Supplementary file 4 [file x-10-x250607-sup4.pdf]

Letter of Response

Dr Matthias Weil
Associate Editor
IUCrData

June 24th, 2025

Re: Submission of revised manuscript reference: wm4230

Dear Dr. Weil,

Thank you very much for your kindness and concern. The manuscript has been revised in accordance with the recommendations. Major changes and additions to the revised manuscript:

Reviewer Comments	Changes
**i) Title**<br>- 'bis' instead of 'tris' (also in the Abstract)	'Bis' has been used instead of 'tris'
**ii) Scheme**<br>- The chemical scheme is not legible and needs to be uploaded in a readable form	The chemical scheme is adjusted for clarity
**iii) Abstract**<br>- the term 'hexadentate' is capable of being misunderstood here (also in the text part). Denticity usually refers to a given (i.e. one) central atom/ion, but in the title compound the oxalate ligands bind to a central atom AND bridge to different ones. Please try to be more precise here (see also iv).<br><br>- it is unclear what you mean with 'non-bonding' K---O contacts. (See also comment in iv))	The term hexadentate has been has been revised to μ6-chelating/ bridging coordination mode<br><br>This has been eliminated
**iv) Structure description**<br>- 'symmetrical planar oxalic acid' Why is this important here?<br><br>- 'that can donate electrons'. I assume you mean 'can coordinate to central atoms/cations'<br><br>- Please give the chemical formulae of the two compounds found in the database search, because this makes the discussion more comprehensible.<br><br>- You wrote: As shown in Fig 1, the Pr^III^ ion is nine-coordinated... However, Fig. 1 shows only	'Symmetrical planar' has been removed<br><br>'… capable of coordinating with metal cations…'<br><br>The chemical formulae of the two compounds are given

eight O ligands. Please revise either the text or the Figure for consistency.	The coordination number of the Pr^{III} ion is nine, and the corresponding Fig. 1 remains unchanged
- The indicated point group symmetry of the mentioned monocapped square antiprism is incorrect because Pr1 is located on a general position which makes the point group symmetry C~1~.	The coordination geometry depicting the arrangement around the metal core for the monocapped square antiprismatic geometry is shown in Fig 2. The C2v point group symmetry has been removed.
- The indicated bonding modes using the 'hapto (or eta)' nomenclature is incorrect because here no pi electrons are present. See: https://goldbook.iupac.org/terms/view/H01881.html Here only \m- and \k-nomenclature is relevant. Please check and revise.	The bonding modes have been corrected per suggestions i.e. μ_4-κ^2O1,O2:κ^2O3,O4:κ^2O1,O3:κ^2O2,O4, μ_4-κO6:κO6':κ^2O5,O6':κ^2O5',O6, and μ_4-κ^2O7,O8:κ^2O7',O8':κ^2O7,O8':κ^2O7',O8
- 'One of the ligands, situated in general position, is...' For simplicity, give atom numbering for this ligand, and also for the ligands situated over inversion centres.	The atom numbering for the ligands is provided.
- You mentioned again the 'non-bonding' K---O interaction. How do you know if it is 'non-bonding'? For argumentation, a bond valence calculation might help here! If it is 'really' non-bonding, its contribution is negligible and thus this statement can be discarded anyway.	The phase of 'non-bonding' K---O contact has been eliminated.
**v) Figures**<br>- Fig. 1: See comment regarding coordination around Pr1 in iv)	Fig. 1 remains unchanged.
- Fig. 2: Is the labelling of axes correct? It seems that the b axis is the shortest whereas it is the longest in reality!	The axes are correctly labeled; thus, no modifications were necessary.
- Fig. 3: Please add the outline of the unit cell.	The outline of the unit cell is added

We hope you find our responses satisfactory and that the manuscript is now appropriate for publication.

Sincerely yours,

Kittipong Chainok, PhD
Associate Professor of Inorganic Chemistry
Thammasat University
